# Serum Phosphorus Is a Fast and Highly Sensitive Marker Predictive of a Complete Cure of Tumor-Induced Osteomalacia

**DOI:** 10.3390/jcm14217870

**Published:** 2025-11-06

**Authors:** Seung Hyun Kim, Young Han Lee, NamKi Hong, Sungjoon Cho, Yumie Rhee

**Affiliations:** 1 Department of Orthopedic Surgery, Severance Hospital, Yonsei University College of Medicine, Seoul 03722, Republic of Korea; sseunghk@yuhs.ac; 2 Department of Radiology, Severance Hospital, Yonsei University College of Medicine, Seoul 03722, Republic of Korea; 3 Department of Internal Medicine, Severance Hospital, Endocrine Research Institute, Yonsei University College of Medicine, Seoul 03722, Republic of Korea

**Keywords:** TIO, PMT, Pi, predictive marker, complete cure

## Abstract

**Background/Objectives:** Tumor-induced osteomalacia (TIO) is a rare acquired paraneoplastic syndrome caused by phosphaturic mesenchymal tumors (PMTs). FGF23, which is overproduced by PMTs, causes hypophosphatemia and osteomalacia, ultimately leading to multiple insufficiency fractures, which are the cause of TIO symptoms. Therefore, recovery from TIO symptoms often takes several months. Due to its paracrine effects, even minuscule amounts of residual PMT can cause treatment to fail. To further compound this, the most confident methods for residual PMTs, serum FGF23 level and ^68^Ga DOTA-based PET/CT, are not readily available. For these reasons, there is currently no established method for early prediction of TIO treatment outcomes after surgery. This study focuses on mineral metabolism and bone turnover markers to identify a clinically practical and readily available biomarker that can predict TIO treatment outcomes. **Methods**: During treatment, we analyzed repeated measurements during treatment of mineral metabolism and bone turnover markers for 19 cases of TIO—Ca, inorganic phosphate (Pi), parathyroid hormone (PTH), 25-hydroxyvitamin D, alkaline phosphatase, Procollagen 1 N-terminal Polypeptide, and β-CrossLaps—in relation to treatment outcomes. We selected predictive marker candidates from among these markers by analyzing their patterns of change during treatment based on three viewpoints—association with (1) cure status, (2) time after treatment, and (3) the interaction effects between (1) and (2) using Linear Mixed Model analysis. We also validated the predictive performance of the selected candidates. **Results:** In long-term follow-up, only serum Pi and PTH levels were significantly associated with all three metrics mentioned above, suggesting that their patterns of change reflect the clinical course and results of TIO treatment. Pi was the only marker that displayed the same associations during short-term follow-up (two weeks and six weeks after treatment), suggesting that it is a rapidly responsive marker. The serum Pi level two weeks after treatment (Odds Ratio = 7.314, *p* = 0.028, AUC value of 0.907) and the normalization of Pi at two weeks post-treatment (Relative Risk = 9.975, *p* = 0.010; sensitivity = 100.0% [95% Confidence Interval (CI) 0.860 to 1.000], specificity = 60.0% [95% CI, 0.208 to 0.600]) were both significantly associated with a complete cure. **Conclusions:** Serum Pi is a fast, simple, and highly sensitive marker that can replace serum FGF23 and ^68^Ga DOTA-based PET/CT in clinical practice for predicting a complete cure of TIO within two weeks of surgery.

## 1. Introduction

Tumor-induced osteomalacia (TIO) is a rare acquired paraneoplastic syndrome which results in bone mineralization defects due to hypophosphatemia caused by phosphaturic mesenchymal tumors (PMTs) [1]. This pathologic condition is caused by tumoral overproduction of fibroblast growth factor 23 (FGF23), which reduces renal phosphate reabsorption by decreasing the expression of NPT2, a Na-P_i_ cotransporter in the proximal tubule and suppresses kidney 1α-hydroxylase, leading to decreased vitamin D activation and impaired Ca absorption [2]. Clinical manifestations of TIO typically include diffuse bone pain due to multiple insufficiency fractures and, less commonly, progressive muscle fatigue and pain, independent of bone pain.

In the past, PMTs were difficult to detect and localize due to their low incidence, small size and varied imaging features [3,4]. Consequently, diagnosis was often delayed for years, leading to serious complications such as multiple insufficiency fractures, progressive disability, and a reduced quality of life. The localization of PMTs is a critical but often challenging step. However, the introduction of systemic venous sampling of FGF23 and ^68^Ga DOTA-based positron emission tomography (PET)/computed tomography (CT) has enabled accurate diagnosis and localization [5,6,7,8].

Burosumab, a human monoclonal antibody against FGF23, has emerged as an effective treatment option. It acts by neutralizing excessive circulating FGF23 and has been reported to successfully improve symptoms and biochemical levels of mineral metabolism and bone turnover markers [9]. However, surgical resection is currently the gold standard for TIO treatment and the only curative modality. Nevertheless, surgical resection faces technical challenges [10]. Soft tissue PMTs have relatively clear margins and can be easily resected, leading to successful results, whereas bone PMTs exhibit ambiguous margins and are difficult to completely resect, leading to unsatisfactory results [11]. Moreover, complete resection of PMTs arising from complex anatomical structures such as the pelvis and hip is much more difficult [12]. Due to the paraneoplastic effects of PMTs, a complete cure cannot be achieved if even only a small amount of tumor remains after surgery.

Common features of mineral metabolism markers in TIOs include persistent hypophosphatemia, low 1,25-dihydroxyvitamin D (1,25(OH)_2_D) levels, normal Ca and 25-hydroxyvitamin D (25(OH)D) levels, and parathyroid hormone (PTH) levels [1,13,14]. These mineral metabolism markers normalize after surgical resection; in particular, inorganic phosphate (Pi) has been reported to recover rapidly [14,15].

Regarding TIO symptoms, bone pain and muscle fatigue can take several months to resolve, whereas bone mineral density (BMD) recovery can take several months to several years [14]. Therefore, it takes a long time to determine whether TIO is completely cured, based on its symptoms to go way. Serum FGF23 level and ^68^Ga DOTA-based PET/CT are currently the most concise methods able to confirm the presence of residual PMTs, yet their use is limited by high cost and requirement for special facilities [16,17]. For this reason, there is currently no suitable testing method to determine whether surgery is successful at an early stage; therefore, patients and their healthcare providers are often unsure whether the surgery is curative. If it has not been curative, waiting for months to find this out can hinder timely treatment.

Complete cure in TIO should be assessed with a comprehensive set of criteria: (1) sustained serum Pi normalization without any phosphate supplement, (2) decline or normalization of serum FGF23 levels, (3) restoration of Pi-regulating parameter, Tubular Reabsorption of Phosphate (TRF)/Tubular maximum for Phosphate reabsorption (TmP)/Glomerular Filtration Rate (GFR) without supplementation, (4) clinical symptom resolution, (5) no evidence of residual PMT on follow-up imaging with ^68^Ga DOTA-based PET/CT. Unfortunately, although serum FGF23 and ^68^Ga DOTA-based PET/CT are the most confident methods for confirming a complete cure of TIO, their practical availability is restricted as they are not routinely performed in most clinical settings.

To develop a new, clinically practical and readily available method for the early identification of complete cure, we focused on mineral metabolism and bone turnover markers (particularly Pi, PTH, 25(OH)D, and ALP), which have been reported to normalize after surgical excision [14]. We analyzed whether changes in these serum markers over the course of treatment and follow-up were associated with a complete cure and evaluated their predictive performance.

## 2. Materials and Methods

### 2.1. Patients

We retrospectively reviewed the medical records of 15 patients who had been treated for TIO between January 2018 and March 2025 at Severance Hospital (Seoul, Korea). This study was approved by the institutional review board (IRB) of Severance Hospital (IRB No. 4-2025-1022). The present study was conducted retrospectively and utilized only electronic records without accessing any patient-derived materials. The study protocol was approved by the IRB, which granted a waiver of informed consent.

**Figure 1:**
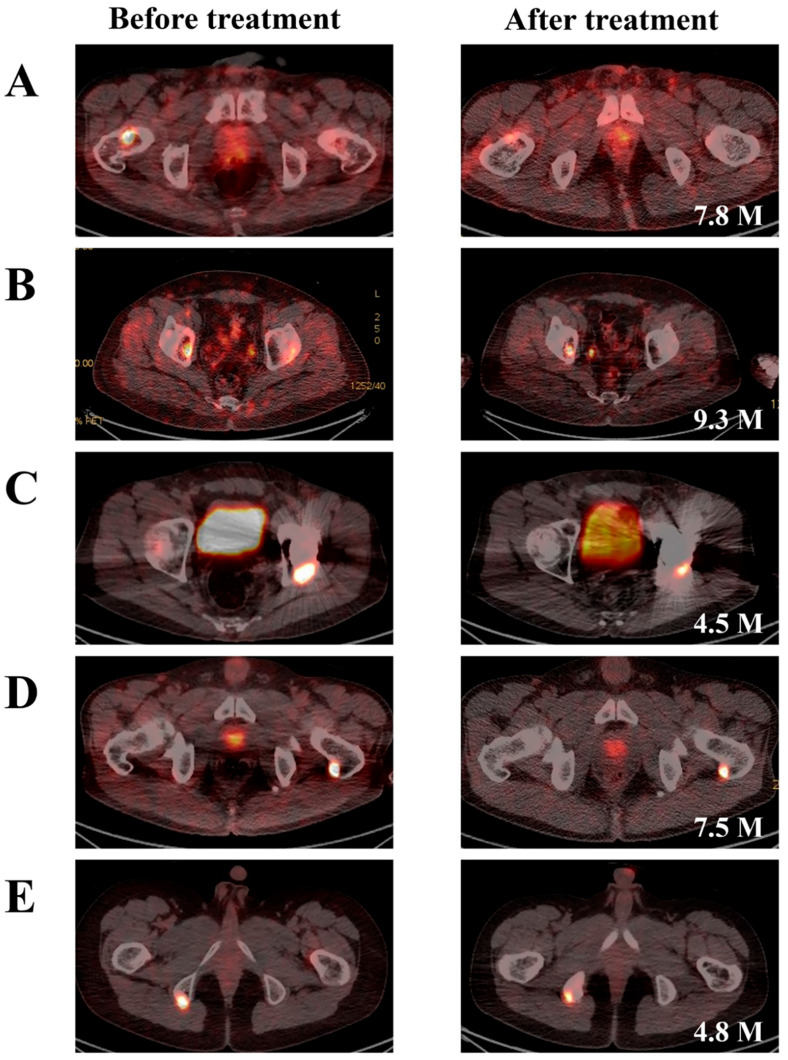
Detection of residual PMTs after first treatment using ^68^Ga-DOTATOC PET/CT. If a patient’s symptoms did not improve, a second ^68^Ga-DOTATOC PET/CT was performed, and if any residual tumor was detected, the treatment was ultimately judged to have failed. The white text in the lower right corner of the right panel indicates the period (in months) after the first treatment. (**A**) patient No. 2; (**B**) patient No. 6; (**C**) patient No. 9; (**D**) patient No. 12; and (**E**) patient No. 13.

Five of the patients did not recover completely after the first treatment, and four of these recovered completely after the second treatment. Therefore, we analyzed the results of 19 treatments performed on 15 patients. All PMTs were diagnosed pathologically using immunostaining against somatostatin receptor 2A (SSTR2A) [5]. In the five patients who did not recover after the first treatment, treatment failure was confirmed by detection of residual PMTs using ^68^Ga-DOTATOC PET/CT (Figure 1); all five patients received subsequent follow-up. Fourteen of the fifteen patients received medication to supplement deficient serum bone metabolites prior to surgery or radiofrequency ablation (RFA) (Table 1). The serum levels of FGF23, Ca, Pi, PTH, 25(OH)D, ALP, P1NP, and CTX were analyzed, and their values at diagnosis are listed in Table 2. The generic names and dosages of the preoperative medication administered to each patient are provided in Table 3.

### 2.2. Measurement of Mineral Metabolism and Bone Turnover Markers

Serum FGF23 levels were measured at diagnosis using an enzyme-linked immunosorbent assay (ELISA) (Intact FGF23 ELISA, Kainos Laboratories, Inc., Tokyo, Japan; reference range, 10–50 pg/mL). Serum Ca, Pi, PTH, 25(OH)D, ALP, P1NP, and β-CrossLaps levels were measured repeatedly throughout the course of treatment from diagnosis to cure. Serum PTH levels were measured using an electrochemiluminescence immunoassay (ECLIA) (Roche Diagnostics; intra-assay coefficients of variation [CV] < 2.5%, inter-assay CV < 5.7%; reference range, 15–65 pg/mL). Serum 25(OH)D levels were measured using a radioimmunoassay (RIA) (INCSTAR Corp; intra-assay CV < 4.1%, inter-assay CV < 7.0%; reference range, >20 ng/mL). Serum ALP levels were measured in international units (IU), and enzyme activity was measured using the p-nitrophenyl phosphate method [18]. Serum procollagen 1 N-terminal polypeptide (P1NP) and β-CrossLaps (CTX) levels were also measured using an ECLIA (P1NP intra-assay CV < 3.6%, P1NP inter-assay CV < 3.9%; reference range, 23–83 ng/mL; Elecsys β-CrossLaps; Roche Diagnostics; CTX intra-assay CV < 3.5%, CTX inter-assay CV < 8.4%; reference range, <0.573 ng/mL).

**Figure 2:**
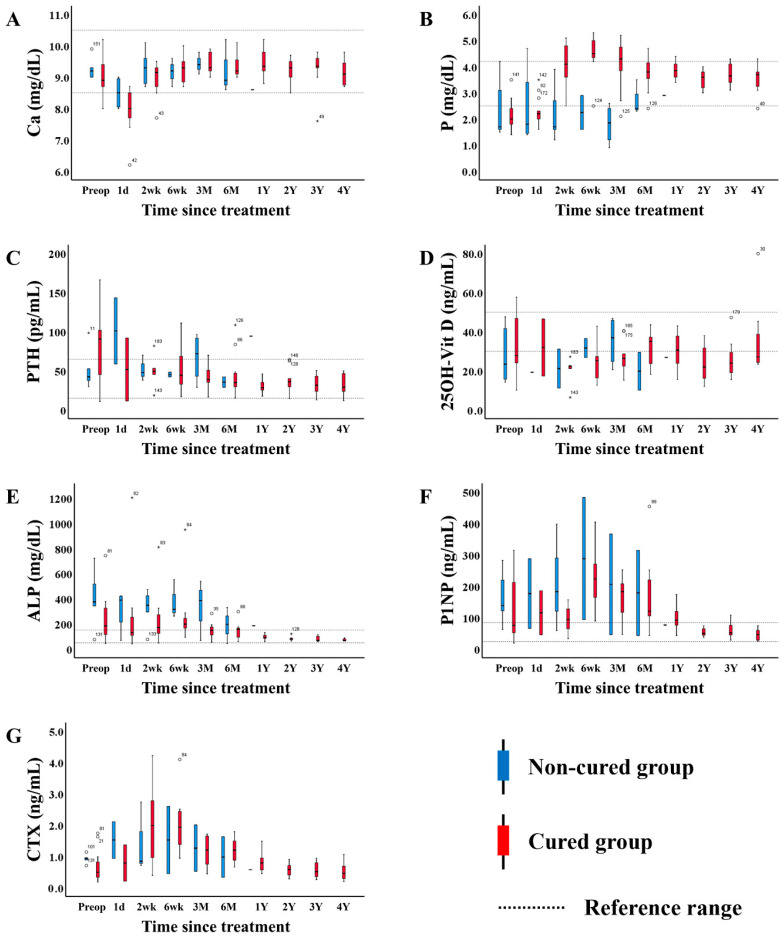
Changes in mineral metabolism and bone turnover markers throughout treatment and follow-up. The data are expressed as the median and interquartile range (IOR) (box). The blue boxes represent the group of patients who were not cured after treatment, and the red boxes represent the group of patients who were cured after treatment. In each panel, the dotted line represents the upper and lower limits of the reference range, and the area between them corresponds to the reference range. Values more than 1.5 × IQR below the first quartile (Q1) or above the third quartile (Q3) are represented by circles and are considered mild outliers. Values more than 3.0 × IQR below Q1 or above Q3 are represented by asterisks and are considered extreme outliers. Superscripts at the upper right of circles and asterisks denote the sample numbers. Since the Non-cured groups were measured and analyzed only up to 6 months, the box-and whisker plots are not shown from 1Y onward in all figures. (**A**) Ca; (**B**) inorganic P; (**C**) PTH; (**D**) 25OH-Vit D; (**E**) ALP; (**F**) P1NP; and (**G**) CTX. Abbreviations: PTH, parathyroid hormone; 25(OH)-Vit D, 25-hydroxyvitamin D; ALP, alkaline phosphatase; P1NP, procollagen 1 N-terminal polypeptide; CTX, β-CrossLaps.

All patients were followed up at the orthopedic outpatient clinic two weeks postoperatively for suture removal and again at six weeks for assessment of the surgical site. Meanwhile, the endocrinology department monitored the mineral metabolism (PTH and 25(OH)D) and bone turnover markers (ALP, P1NP, and CTX) every two to three months for six months following treatment. Ca and Pi levels were measured at all follow-up visits, as they are included in routine chemistry panels. Therefore, there were few missing values in the short-term follow-up data (Table 4, Table 5 and Table 6, within 3 months postoperatively), the number of missing values increased in the long-term follow-up data (Figure 2, up to four years). Horizontal box-and-whisker plots were generated after excluding missing values. The median, interquartile range (IQR), and whiskers were calculated based on available data, and outliers were indicated as individual points.

### 2.3. Statistical Analysis

To compare the changes in mineral metabolism and bone turnover markers between cured and non-cured groups throughout the treatment course, repeatedly measured marker data are displayed as horizontal box-and-whisker plots. A linear Mixed Model (LMM) was used to determine whether changes in any bone metabolism marker throughout the treatment course were associated with the treatment, complete cure, and the interaction effects of the treatment and a complete cure. In this model, each mineral metabolism and bone turnover maker was treated as a dependent variable, and with time, complete cure, and their interaction (complete cure * time since treatment) were included as fixed effects. A random intercept was specified for each subject to account for baseline differences. The associations between serum Pi and PTH levels and a complete cure were analyzed using logistic regression analysis. The area under the curve (AUC) values predicting a complete cure were calculated from the receiver operating characteristic (ROC) curve, and cutoff values were derived based on the Youden index. The χ^2^ test and Fisher’s extract test (when necessary) were used to analyze the association between normalization of serum Pi and PTH levels and a complete cure, and predictive performance was validated using two-way contingency table analysis. Statistical analyses were performed using SPSS (version 26.0, SPSS, Inc., Chicago, IL, USA). All *p*-values were two-tailed and a *p*-value < 0.05 was considered significant.

## 3. Results

### 3.1. Patient Demographics and PMT Characteristics

The mean age of the 15 patients was 44.6 years, with 10 males and 5 females. The mean period from symptom onset to diagnosis was 4.1 years. Fourteen patients experienced multiple insufficiency fractures. Out of fifteen PMTs, two originated from soft tissue, two from soft tissue and bone, and eleven from bone. Nine PMTs were located around the pelvis and hip joint, and six PMTs were located in the lower extremities. The average PMT size of the was 5.77 cm^3^ (Table 1).

The first treatment attempts involved surgical resection (thirteen cases) and RFA (two cases). Five patients did not achieve a complete cure at this stage (three cases of surgical resection and two cases of RFA). The residual PMTs of these patients, all of which occurred around the pelvis and hip, were detected and confirmed using ^68^Ga-DOTATOC PET/CT (Figure 1). Case A, B, D, and E achieved a complete cure following second surgery, while case C has not yet received further surgical treatment. The mean follow-up period until secondary treatment was 11.38 months. The mean follow-up period for the 15 patients was 43.37 months. This study analyzed a total of nineteen cases from fifteen patients, including four cases with secondary surgery. Of the 17 surgeries, including secondary surgery, seven were intralesional surgery (curettage) and ten were En bloc recession (Table 1).

### 3.2. Changes in Mineral Metabolism and Bone Turnover Markers During Treatment of TIO

The serum levels of FGF23, Ca, Pi, PTH, 25(OH)D, ALP, P1NP, and CTX were analyzed, and their values at diagnosis are listed in Table 2. Serum Ca was within the normal range. Serum FGF23 and PTH were elevated. The bone formation markers ALP and P1NP and the bone absorption marker CTX were also increased, indicating an increased bone turnover rate. Conversely, Pi and 25(OH)D had decreased, with FGF23 increased by the PMT. Preoperative medication to supplement each patients’ deficient Pi and 25(OH)D levels are also summarized in Table 3. In all patients, preoperative medications were discontinued immediately prior to surgical resection.

To monitor changes in bone metabolism markers throughout treatment and follow-up, we repeatedly measured patient data, which are displayed as horizontal box-and-whisker plots (Figure 2). The blue boxes represent the “non-cured group”, and the red boxes represent the “cured group”. Serum Ca remained stable within the reference range throughout treatment and follow-up in both groups. Overall, all mineral metabolism and bone turnover markers, excluding 25(OH)D, eventually returned to their reference ranges during long-term follow-up (more than two years). Among those markers that were increased at diagnosis, PTH and ALP showed faster recovery in the cured group than in the non-cured group, but no such difference was observed for P1NP and β-CrossLaps. Serum Pi and 25(OH)D, which were abnormally low at diagnosis, showed significantly different patterns. Pi recovered very quickly after treatment in the cured group, but 25(OH)D did not recover even after long-term follow-up. Pi, PTH, and ALP recovered after surgical resection and showed differences in recovery rates between the cured group and non-cured groups, suggesting that their potential utility for predictive markers for the complete cure of TIO.

### 3.3. Candidate Predictive Markers for Complete Cure of TIO

For a new predictive marker to be successfully introduced into clinical practice, it should be associated with treatment results and be useful for postoperative surveillance. Therefore, we analyzed data on repeated measurements of mineral metabolism and bone turnover markers from diagnosis to 3-month follow-up after treatment. Linear Mixed Model (LMM) analysis is a suitable method of identifying factors associated with treatment process and outcome from data measured repeatedly during treatment and follow-up [19]. We used LMM analysis to analyze the change patterns of each serum bone metabolism marker based on three viewpoints: (1) association with complete cure status, (2) association with time elapsed since treatment, and (3) association with the interaction effects between complete cure status and time elapsed since treatment (Table 4). Only Pi and PTH showed significant associations with all three viewpoints, suggesting that their patterns of change reflect the clinical outcomes of TIO treatment and indicating their potential as predictive markers for the complete cure of TIO.

An ideal predictive marker responds quickly to treatment and can predict outcomes within a short period of time since treatment. To verify the suitability of Pi and PTH as markers, we analyzed their patterns of change during short-term follow-up (two weeks and six weeks after treatment) using LMM analysis (Table 5). Only Pi showed significant associations with all three viewpoints mentioned above at both two and six weeks after treatment, demonstrating that serum Pi is a very useful predictive marker for a complete cure of TIO. On the contrary, the change in PTH during the short-term follow-up did not reflect the clinical course or outcomes of the TIO treatment.

Recently, large scale-studies have been reported highlighting the significance of serum Pi changes in the treatment course of TIO. In a retrospective study of 117 TIO patients, serum Pi levels were normalized within seven days in 82.7% of those who achieved a cure following tumor resection [14]. A retrospective study involving 230 TIO patients reported that preoperative serum Pi levels were a predictive marker for successful treatment, with an AUC of 0.6465 [20]. These findings support the potential of postoperative changes in serum Pi as a valuable marker for predicting complete cure in TIO.

### 3.4. Predictive Performance of Pi for Complete Cure of TIO

The predictive performance of Pi was analyzed in relation to both continuous variables (measurements of serum Pi) and categorical variables (within reference range or not). For continuous variables, we applied logistic regression. Only serum Pi levels at two weeks after treatment were associated with a complete cure (odds ratio [OR] = 7.314, *p* = 0.028), with an AUC value of 0.907. The cutoff value derived from the Youden index was 3.6 mg/dL, with a sensitivity of 78.6% and a specificity of 80.0% (Table 6). For categorical variables, we analyzed the results of the χ^2^ test and two-way contingency table analysis. Only normalization of Pi at two weeks after treatment was associated with a complete cure (relative risk [RR] = 9.975, *p* = 0.010), with a sensitivity of 100.0% (95% Confidence Interval [CI], 0.860 to 1.000), a specificity of 60.0% (95% CI, 0.208 to 0.600), a positive predictive value (PPV) of 87.5% (95% CI, 0.753 to 0.875), and a negative predictive value (NPV) of 100.0% (95% CI, 0.347 to 1.000) (Table 6). These analyses suggest that the Pi serum level at two weeks after treatment is a highly sensitive predictive marker for the complete cure of TIO.

Measurement of serum Pi is inexpensive and easily performed in practice, and it can accurately and rapidly predict the treatment outcomes of TIO just at two weeks after treatment. Therefore, serum Pi is an excellent, cost-effective predictor of TIO treatment outcomes.

## 4. Discussion

TIO is clinically characterized by hypophosphatemia due to excessive production of FGF23 by PMTs. This inhibits the expression of NPT2a/NPT2c and the activity of 1α-hydroxylase, leading to impaired Pi reabsorption, decreased 1,25(OH)_2_D, and consequently osteomalacia [2]. The symptoms of TIO are often nonspecific including bone pain derived from multiple insufficiency fractures and muscle fatigue/weakness, leading to a delayed diagnosis. In this cohort, the average delay in diagnosis was 4.1 years (range: 1 to 15 years). Misdiagnosis is also common. Symptom-wise, it may be confused with osteoporosis, rheumatism, or other musculoskeletal disorders, and in terms of laboratory blood testing, it may be confused with diseases that cause hyperparathyroidism. As a real-world example, patient 4, a 69-year-old man with the longest diagnostic delay in this study, 15 years, had a history of parathyroidectomy due to a misdiagnosis of brown tumor.

Since the introduction of systemic venous sampling of FGF23 and ^68^Ga DOTA-based PET/CT in the management of TIO, diagnosis has become easier and localization of PMTs more feasible, resulting in significantly improved clinical outcomes, with a symptom improvement rate of 97.6% [21] and a complete cure rate of 83.8% [14]. Surgical resection and RFA were reported to have similar outcomes [22]. Beyond treatment modality (surgical resection or RFA), a more important consideration for complete resection is the anatomical localization of the PMT. PMTs around the pelvis and hip are difficult to completely resect [22]. In this study, the complete cure rate was 66.7% (10 out of 15 patients) at first treatment, and the final complete cure rate, including reoperation, was 93.3% (14 out of 15 patients). In all five cases (three cases of surgical resection and two cases of RFA) for which the first treatment failed, the PMTs were located around the pelvis and hip. Four of these patients were completely cured through reoperation. One patient had a residual PMT, as confirmed by ^68^Ga DOTATOC PET/CT, but his symptoms improved with supplemental medication and the second operation was postponed.

Localization of PMTs is essential for the complete cure of TIO. PMTs are reported to exhibit low attenuation on T1-weighted images and high signal intensity on T2-weighted and Short Tau Inversion Recovery (STIR) sequences on MRI [3,4]. Although MRI is essential for defining the anatomical margins of a PMT and planning surgical margins, its role is limited in the localization of PMTs at diagnosis and the detection of residual PMTs after surgical resection [23]. In clinical practice, ^68^Ga DOTA-based PET/CT is the only imaging modality that provides substantial aid in the localization of PMTs at diagnosis and the detection of residual PMTs after surgical resection [6]. Because ^68^Ga DOTA-based PET/CT has high operating and radiopharmaceutical production costs [16] and long-term risks associated with radiation exposure [17], it is not readily available for clinical use. Therefore, despite its high sensitivity and specificity for PMTs, ^68^Ga DOTA-based PET/CT is not suitable for early prediction of TIO treatment outcomes.

Mineral metabolism and bone turnover markers in TIO have been well characterized. According to a systemic review of 769 articles, hypophosphatemia (99.8%), increased FGF23 (95.5%), increased ALP (94.9%), and decreased 1,25(OH)_2_D (69.8) were observed at TIO diagnosis, and serum Pi and FGF23 levels were normalized in approximately 91.5% and 81.4% of patients, respectively [21]. A large cohort study of 117 patients reported rapid recovery of serum Pi after surgery. The postoperative cumulative recovery rate was 28.6% on days 1 to 3, 79.7% on days 4 to 7, and 95.0% on day 14 postoperatively [14]. The current recommendation involves frequent measurement of serum Pi until it normalizes after surgery and monitoring if recurrence is suspected [24]. TIO is a disease based on phosphate metabolism, and serum Pi level monitoring is an essential component of intervention from diagnosis to treatment evaluation and prognosis.

The limitations of this study should be considered. First, it is a retrospective study with a relatively small cohort of 19 cases treated at a single institute. Second, we were unable to measure the changes in serum levels of FGF23, undoubtedly the most critical pathophysiological mediator of TIO and 1,25(OH)2D, the most specific active vitamin D to TIO throughout the treatment course.

## 5. Conclusions

Serum Pi is a rapid, simple, and highly sensitive marker for predicting the complete cure of TIO. The predictive performance of serum Pi level at two weeks after treatment had an AUC value of 0.907 (OR = 7.314) and normalization of Pi at two weeks after treatment was also associated with a complete cure (Relative Risk = 9.975, *p* = 0.010), with a sensitivity of 100.0% (95% 0.860 to 1.000), and a specificity of 60.0% (95% CI, 0.208 to 0.600). These results demonstrate that serum Pi level is an excellent predictor of a complete cure of TIO and can be readily used clinically as an alternative to serum FGF23 level and ^68^Ga DOTA-based PET/CT.

## Figures and Tables

**Table 1:** Clinical characteristics of patients and tumors.

Case No.	Patient No.	Sex	Age (Years)	Duration (Years)	Multiple Insufficiency Fractures ^1^	Tissue Involved	Tumor Location	Tumor Size (cm^3^) ^2^	1st Treatment Procedure	2nd Treatment Procedure	Interval Until 2nd Treatment (Months)	Follow-Up Period (Months)	Cure
1	1	M	52	2	Yes	Bone	fibula head	3.77	Surgery ^3^			9.6	Yes
2	2	M	59	1.5	Yes	Bone and Soft	femur neck	0.92	Surgery ^4^	Surgery ^4^		47.8	No
3 ^5^											9.5		Yes
4	3	F	31	1	Yes	Bone	femur head	1.97	Surgery ^3^			73.1	Yes
5	4	M	69	15	Yes	Bone	femur head	22.61	Surgery ^4^			60.0	Yes
6	5	F	58	1.5	Yes	Soft tissue	adjacent to GT tip	1.83	Surgery ^4^			55.1	Yes
7	6	M	44	3.3	Yes	Bone	acetabulum	3.14	RFA	Surgery ^3^		55.5	No
8 ^5^											20.5		Yes
9	7	M	31	7	Yes	Bone	distal femur	20.76	Surgery ^3^			41.8	Yes
10	8	M	24	2	Yes	Bone	tibia plateau	0.68	Surgery ^3^			76.0	Yes
11	9	M	54	3.5	Yes	Bone	acetabulum	2.64	Surgery ^3^		-	2.7	No
12	10	F	54	5	Yes	Soft tissue	2nd toe	0.69	Surgery ^4^			34.7	Yes
13	11	F	25	6	Yes	Bone	proximal tibia	0.46	Surgery ^4^			38.7	Yes
14	12	M	63	8	No	Bone	GT tip	0.55	RFA	Surgery ^4^		48.3	No
15 ^5^											9.8		Yes
16	13	M	20	1.5	Yes	Bone	ischial tuberosity	2.03	Surgery ^3^	Surgery ^3^		31.3	No
17 ^5^											5.7		Yes
18	14	M	52	1.8	Yes	Bone and Soft	distal tibia	1.16	Surgery ^3^			73.0	Yes
19	15	F	33	3	Yes	Bone	PSIS	23.27	Surgery ^4^			2.9	Yes
Mean ± SD (min–max)			44.6 ± 15.9 (20–69)	4.1 ± 3.7 (1–15)				5.77 ± 8.58 (0.46–23.27)			11.38 ± 6.36 (5.7–20.5)	43.37 ± 24.07 (2.7–76.0)	

^1^ multiple fractures without definite trauma history, detected by WBBS as hot uptake of 99mTc-HDP in the ribs, spine, or pelvis. ^2^ calculated using the ellipsoid formula, ^3^ intralesional surgery (curettage), ^4^ En bloc resection, ^5^ secondary surgical treatment. Abbreviations: GT, Greater Trochanter of femur; RFA, radiofrequency ablation; PSIS, Posterior Superior Iliac Spine; WBBS, Whole Body Bone Scan; 99mTc-HDP, Technetium-99m Hydroxymethylene diphosphonate; SD, Standard Deviation.

**Table 2:** Serum levels of bone metabolism and bone turnover markers at diagnosis.

Case No.	Mineral Metabolism Marker					Bone Turnover Marker		
						Bone Formation		Bone Resorption
	FGF23 (pg/mL)	Ca (mg/dL)	Pi (mg/dL)	PTH (pg/mL)	25(OH)D (ng/mL)	ALP (IU/L)	P1NP (ng/mL)	CTX (ng/mL)
1	163.88	8.7	2.8	81.8	29.440	358.0	196.0	1.760
2	252.14	9.0	1.7	112.1	11.220	451.0	220.0	0.958
3 ^1^	50.90	8.9	2.4	42.2	29.440	331.0	315.0	1.630
4	159.04	8.7	1.7	101.8	27.420	272.0	62.3	0.375
5	3832.00	8.3	2.2	10.8	22.180	92.0	36.0	0.208
6	182.72	8.9	1.9	109.2	27.290	142.0	74.4	0.339
7	-	9.0	3.1	52.6	23.370	376.0	139.0	0.889
8 ^1^	235.00	8.7	2.1	90.0	29.290	173.0	61.0	0.429
9	4244.70	9.4	1.8	98.9	25.240	745.0	245.0	1.740
10	699.50	8.6	2.8	52.9	47.410	130.0	88.5	0.708
11	163.20	9.2	1.5	29.6	41.600	344.0	122.0	1.140
12	47.41	8.4	2.0	70.1	57.620	83.0	19.4	0.083
13	124.99	9.4	1.8	166.2	24.040	196.0	76.7	0.568
14	1676.90	9.3	4.2	42.3	14.170	75.0	62.5	0.709
15 ^1^	-	10.2	3.5	28.9	10.140	45.0	43.0	0.334
16	314.78	9.9	1.6	37.7	47.600	724.0	283.0	0.939
17 ^1^	-	9.4	1.4	96.5	46.770	379.0	-	-
18	210.65	8.0	2.0	91.2	47.420	237.0	193.0	0.987
19	303.38	8.9	2.7	86.4	22.090	116.0	-	-
Mean ± SD	791.32 ± 1328.43	9.00 ± 0.54	2.27 ± 0.74	73.75 ± 38.08	30.724 ± 13.589	277.32 ± 202.01	131.58 ± 92.73	0.812 ± 0.52

^1^ Secondary surgical treatment. Serum level color: blue, below reference range; black, within reference range; red, above reference range. Abbreviations: PTH, parathyroid hormone; 25(OH)D, 25-hydroxyvitamin D; ALP, alkaline phosphatase; IU, international unit; P1NP, procollagen 1 N-terminal polypeptide; CTX, β-CrossLaps.

**Table 3:** Preoperative PO medications to supplement mineral metabolism markers.

Case No.	Elemental Ca (mg/Day)	Elemental Phosphorus (mg/Day)	Vitamin D Supplements		
			1,25(OH)_2_D (μg/Day)	Cholecalciferol (mg/Day)	Alfacalcidol (μg/Day)
1	100	3000	250	10	
2	200	3000	500	20	
3 ^1^	100	-	250	10	
4	100	3000	500	10	
5	2100	750	500	10	
6	100	2250	500	10	
7	-	1500	-	-	1.5
8 ^1^	100	2250	-	10	1.5
9	200	3000	500	20	
10	100	2250	500	10	
11	100	2250	500	10	
12	100	2250	750	10	
13	100	3000	750	10	
14	-	-	-	-	
15 ^1^	-	-	-	-	
16	200	2250	1000	20	
17 ^1^	200	2500	1250	20	
18	100	4000	-	10	1.5
19	-	1000	-		1.0

^1^ secondary surgical treatment. Abbreviations: PO, Per Os; 1,25(OH)_2_D, 1,25-dihydroxyvitamin D.

**Table 4:** LMM analysis for mineral metabolism and bone turnover markers throughout treatment and follow-up ^1^.

	Mineral Metabolism Marker								Bone Turnover Marker					
									Bone Formation				Bone Resorption	
	Ca		Pi		PTH		25(OH)D		ALP		P1NP		CTX	
	E ± SE (95% CI)	*p*	E ± SE (95% CI)	*p*	E ± SE (95% CI)	*p*	E ± SE (95% CI)	*p*	E ± SE (95% CI)	*p*	E ± SE (95% CI)	*p*	E ± SE (95% CI)	*p*
**Intercept**	8.10 ± 0.16 (7.77 to 8.42)	<*0.000*	1.63 ± 0.26 (1.11 to 2.15)	<*0.000*	91.79 ± 10.10 (71.36 to 112.21)	<*0.000*	31.63 ± 3.65 (24.23 to 39.03)	<*0.000*	246.60 ± 58.43 (123.79 to 369.40)	*0.001*	89.10 ± 28.48 (29.61 to 148.58)	*0.005*	0.66 ± 0.25 (0.14 to 1.18)	*0.015*
**Complete cure**	0.73 ± 0.32 (0.09 to 1.37)	*0.027*	1.03 ± 0.51 (0.01 to 2.05)	*0.047*	−40.15 ± 19.56 (−79.80 to −0.50)	*0.047*	−6.55 ± 7.12 (−20.97 to 7.87)	*0.363*	131.00 ± 114.20 (−108.87 to 370.87)	*0.266*	78.97 ± 53.93 (−33.71 to 191.65)	*0.159*	0.36 ± 0.48 (−0.63 to 1.35)	*0.456*
**Time since treatment**	0.25 ± 0.05 (0.15 to 0.35)	<*0.000*	0.58 ± 0.08 (0.41 to 0.75)	<*0.000*	−10.35 ± 2.65 (−15.83 to −4.87)	*0.001*	−1.20 ± 0.92 (−3.11 to 0.70)	*0.204*	−6.14 ± 7.35 (−21.51 to 9.24)	*0.414*	23.15 ± 7.46 (7.64 to 38.65)	*0.005*	0.23 ± 0.09 (0.05 to 0.41)	*0.015*
Complete cure * **Time since treatment ^2^**	−0.16 ± 0.09 (−0.35 to 0.03)	*0.104*	−0.75 ± 0.16 (−1.07 to −0.42)	<*0.000*	12.83 ± 5.22 (2.02 to 23.64)	*0.022*	2.67 ± 1.82 (−1.10 to 6.43)	*0.156*	−7.69 ± 14.22 (−37.53 to 22.14)	*0.595*	−20.04 ± 15.76 (−52.66 to 12.58)	*0.216*	−0.20 ± 0.19 (−0.60 to 0.19)	*0.302*

^1^ analyzed by fixed model. ^2,^* interaction effects between Complete cure and Time since treatment. Abbreviations: LMM, linear mixed model; PTH, parathyroid hormone; 25(OH)D, 25-hydroxyvitamin D; ALP, alkaline phosphatase; P1NP, procollagen 1 N-terminal polypeptide; CTX, β-CrossLaps; E, estimate; SE, standard error; CI, confidence interval.

**Table 5:** LMM analysis for serum Pi and PTH during short-term follow-up ^1^.

	Pi				PTH			
	2 wk		6 wk		2 wk		6 wk	
	E ± SE (95% CI)	*p*	E ± SE (95% CI)	*p*	E ± SE (95% CI)	*p*	E ± SE (95% CI)	*p*
**Intercept**	0.95 ± 0.31 (0.30 to 1.59)	*0.007*	1.09 ± 0.27 (0.51 to 1.66)	*0.001*	100.40 ± 14.01 (70.93 to 129.86)	<*0.000*	93.09 ± 11.65 (69.44 to 116.75)	<*0.000*
**Complete cure**	1.61 ± 0.60 (0.34 to 2.87)	*0.016*	1.41 ± 0.54 (0.28 to 2.54)	*0.017*	−41.28 ± 26.530 (−97.30 to 14.75)	*0.138*	−31.90 ± 22.89 (−78.41 to 14.61)	*0.172*
**Time since treatment**	0.93 ± 0.130 (0.66 to 1.21)	<*0.000*	0.87 ± 0.11 (0.64 to 1.10)	<*0.000*	−17.47 ± 6.84 (−33.64 to −1.30)	*0.038*	−11.31 ± 3.89 (−19.41 to −3.20)	*0.009*
Complete cure * **Time since treatment ^2^**	−1.03 ± 0.25 (−1.57 to−0.50)	*0.001*	−0.94 ± 0.22 (−1.39 to −0.49)	<*0.000*	17.39 ± 12.22 (−12.28 to 47.06)	*0.203*	8.280 ± 8.12 (−8.61 to 25.17)	*0.320*

^1^ analyzed by fixed model. ^2,^* interaction effects between Complete cure and Time since treatment. Abbreviations: LMM, linear mixed model; PTH, parathyroid hormone; wk, week; E, estimate; SE, standard error; CI, confidence interval; * indicates the interaction between “complete cure” and “time since treatment”.

**Table 6:** The predictive performance of Pi for complete cure of TIO.

		Continuous Variable							Categorical Variable: Contingency Table Analysis					
		Logistic Regression		ROC Curve		Youden Index			χ^2^ Square Test		Diagnostic Test			
		OR (95% CI)	*p*	AUC (95% CI)	*p*	Cutoff value (mg/dL, pg/mL)	Sen	1-Spe	Pearson χ^2^	*p* ^1^	Sen (%) (95% CI)	Spe (%) (95% CI)	PPV (%) (95% CI)	NPV (%) (95% CI)
**Pi**	**2 wk**	7.314 (1.239 to 43.175)	*0.028*	0.907 (0.750 to 1.000)	*0.008*	3.6	0.786	0.200	9.975	*0.010*	100.0 (0.860 to 1.000)	60.0 (0.208 to 0.600)	87.5 (0.753 to 0.875)	100.0 (0.347 to 1.000)
	**6 wk**	9.127 (0.378 to 220.654)	*0.174*	0.929 (0.741 to 1.000)	*0.079*	4.2	0.857	0.000	3.938	*0.222*	100.0 (0.865 to 1.000)	50.0 (0.028 to 0.500)	87.5 (0.757 to 0.875)	100.0 (0.056 to 1.000)
	**3 M**	16.894 (0.426 to 669.677)	*0.132*	0.955 (0.850 to 1.000)	*0.009*	2.7	0.909	0.000	7.111	*0.027*	91.7 (0.752 to 0.993)	75.0 (0.256 to 0.978)	91.7 (0.752 to 0.993)	75.0 (0.256 to 0.978)
**PTH**	**2 wk**	0.992 (0.913 to 1.077)	*0.840*	0.444 (0.020 to 0.869)	*0.796*	47.7	0.333	0.667	0.321	*1.000*	83.3 (0.676 to 0.991)	33.3 (0.018 to 0.6484)	71.4 (0.579 to 0.849)	50.0 (0.027 to 0.972)
	**6 wk**	1.013 (0.944 to 1.086)	*0.722*	0.500 (0.138 to 0.862)	*1.000*	45.1	0.500	0.500	0.625	*1.000*	75.0 (0.750 to 0.930)	0.0 (0.000 to 0.718)	75.0 (0.750 to 0.930)	0.0 (0.000 to 0.718)
	**3 M**	0.943 (0.881 to 1.009)	*0.088*	0.750 (0.406 to 1.000)	*0.157*	41.1	0.700	0.250	2.715	*0.176*	90.0 (0.741 to 0.994)	50.0 (0.103 to 0.736)	81.8 (0.674 to 0.904)	66.7 (0.137 to 0.982)

^1^ Fisher’s exact, two-tailed *p*. Abbreviations: TIO, tumor-induced osteomalacia; ROC, receiver operating characteristic; OR, odds ratio; AUC, area under the curve; Sen, sensitivity; Spe, specificity; PPV, positive predictive value; NPV, negative predictive value; wk, week; M, month.

## Data Availability

The data presented in this study are available on request from the corresponding author.

## References

[1] Rendina D., Abate V., Cacace G., D’Elia L., De Filippo G., Del Vecchio S., Galletti F., Cuocolo A., Strazzullo P. (2022). Tumor-induced Osteomalacia: A Systematic Review and Individual Patient’s Data Analysis. J. Clin. Endocrinol. Metab..

[2] Jüppner H. (2011). Phosphate and FGF-23. Kidney Int. Suppl..

[3] Broski S.M., Folpe A.L., Wenger D.E. (2019). Imaging features of phosphaturic mesenchymal tumors. Skelet. Radiol..

[4] Gupta A., Kandasamy D., Sharma R., Damle N., Goyal A., Goyal A., Agarwal S., Dharmashaktu Y. (2023). Imaging characteristics of phosphaturic mesenchymal tumors. Acta Radiol..

[5] Lee S., Hong N., Shin S., Kim S.I., Yun M., Kim S.K., Rhee Y. (2022). Diagnostic Utility of Somatostatin Receptor 2A Immunohistochemistry for Tumor-induced Osteomalacia. J. Clin. Endocrinol. Metab..

[6] Kato A., Nakamoto Y., Ishimori T., Hayakawa N., Ueda M., Temma T., Sano K., Shimizu Y., Saga T., Togashi K. (2021). Diagnostic performance of (68)Ga-DOTATOC PET/CT in tumor-induced osteomalacia. Ann. Nucl. Med..

[7] Zhang J., Zhu Z., Zhong D., Dang Y., Xing H., Du Y., Jing H., Qiao Z., Xing X., Zhuang H. (2015). 68Ga DOTATATE PET/CT is an Accurate Imaging Modality in the Detection of Culprit Tumors Causing Osteomalacia. Clin. Nucl. Med..

[8] Lee J.-Y., Park H.-S., Han S., Lim J.K., Hong N., Park S.I., Rhee Y. (2017). Localization of Oncogenic Osteomalacia by Systemic Venous Sampling of Fibroblast Growth Factor 23. Yonsei Med. J..

[9] de Beur S.M.J., Miller P.D., Weber T.J., Peacock M., Insogna K., Kumar R., Rauch F., Luca D., Cimms T., Roberts M.S. (2021). Burosumab for the Treatment of Tumor-Induced Osteomalacia. J. Bone Miner. Res..

[10] Dahir K., Zanchetta M.B., Stanciu I., Robinson C., Lee J.Y., Dhaliwal R., Charles J., Civitelli R., Roberts M.S., Krolczyk S. (2021). Diagnosis and Management of Tumor-induced Osteomalacia: Perspectives From Clinical Experience. J. Endocr. Soc..

[11] Sun Z.-J., Jin J., Qiu G.-X., Gao P., Liu Y. (2015). Surgical treatment of tumor-induced osteomalacia: A retrospective review of 40 cases with extremity tumors. BMC Musculoskelet. Disord..

[12] Liu S., Zhou X., Liang A., Xing J., Liu Y., Jin J., Zhang J., Xia W. (2024). Orthopedic Surgical Treatment of Patients with Tumor-induced Osteomalacia Located in the Hip Bones: A Retrospective Analysis of 10 Years in a Single Center. Orthop. Surg..

[13] Hidaka N., Koga M., Kimura S., Hoshino Y., Kato H., Kinoshita Y., Makita N., Nangaku M., Horiguchi K., Furukawa Y. (2022). Clinical Challenges in Diagnosis, Tumor Localization and Treatment of Tumor-Induced Osteomalacia: Outcome of a Retrospective Surveillance. J. Bone Miner. Res..

[14] Shan C., Wei Z., Li S., Zhang Z., Yue H., Yu W., Yang Q., Zhang Z. (2025). Postoperative outcome and clinical management of tumor-induced osteomalacia: A single-center retrospective cohort study on 117 patients. Osteoporos. Int..

[15] Wang H., Zhong D., Liu Y., Jiang Y., Qiu G., Weng X., Xing X., Li M., Meng X., Li F. (2015). Surgical Treatments of Tumor-Induced Osteomalacia Lesions in Long Bones: Seventeen Cases with More Than One Year of Follow-up. J. Bone Jt. Surg. Am..

[16] Sipos D., Debreczeni-Máté Z., Ritter Z., Freihat O., Simon M., Kovács Á. (2024). Complex Diagnostic Challenges in Glioblastoma: The Role of ^18^F-FDOPA PET Imaging. Pharmaceuticals.

[17] Masselli G., Casciani E., De Angelis C., Sollaku S., Gualdi G. (2021). Clinical application of (18)F-DOPA PET/TC in pediatric patients. Am. J. Nucl. Med. Mol. Imaging.

[18] Tietz N.W., A Burtis C., Duncan P., Ervin K., Petitclerc C.J., Rinker A.D., Shuey D., Zygowicz E.R. (1983). A reference method for measurement of alkaline phosphatase activity in human serum. Clin. Chem..

[19] Kim S.H., Shin K., Moon S., Jang J., Kim H.S., Suh J., Yang W. (2017). Reassessment of alkaline phosphatase as serum tumor marker with high specificity in osteosarcoma. Cancer Med..

[20] Li X., Jiang Y., Huo L., Wu H., Liu Y., Jin J., Yu W., Lv W., Zhou L., Xia Y. (2020). Nonremission and Recurrent Tumor-Induced Osteomalacia: A Retrospective Study. J. Bone Miner. Res..

[21] Álvarez-Rivas N., Lugo-Rodríguez G., Maneiro J.R., Iñiguez-Ubiaga C., Melero-Gonzalez R.B., Iglesias-Cabo T., Carmona L., García-Porrúa C., de Toro-Santos F.J. (2024). Tumor-induced osteomalacia: A systematic literature review. Bone Rep..

[22] Memon S.S., Patel M.A., Lila A., Jadhav S., Sarathi V., Karlekar M., Barnabas R., Patil V., Kulkarni S., Rathod K. (2024). Long-Term Follow-Up Data of Tumor-Induced Osteomalacia Managed with Surgery and/or Radiofrequency Ablation from a Single Center. Calcif. Tissue Int..

[23] Rayamajhi S.J., Yeh R., Wong T., Dumeer S., Mittal B.R., Remotti F., Chikeka I., Reddy A.K. (2019). Tumor-induced osteomalacia—Current imaging modalities and a systematic approach for tumor localization. Clin. Imaging.

[24] de Beur S.M.J., Minisola S., Xia W., Abrahamsen B., Body J., Brandi M.L., Clifton-Bligh R., Collins M., Florenzano P., Houillier P. (2023). Global guidance for the recognition, diagnosis, and management of tumor-induced osteomalacia. J. Intern. Med..

